# Leveraging biosensors in clinical and research settings: a guide to device selection

**DOI:** 10.1038/s44277-025-00037-w

**Published:** 2025-07-02

**Authors:** Lana Ruvolo Grasser

**Affiliations:** 1 Department of Psychology, College of Liberal Arts and Sciences, Wayne State University, Detroit, MI USA; 2 Ben L. Silberstein Institute for Brain Health, Division of Research and Innovation, Wayne State University, Detroit, MI USA

**Keywords:** Autonomic nervous system, Biomarkers

## Abstract

Psychophysiological variables—e.g., heart rate (HR), heart rate variability (HRV), and skin conductance response (SCR)—reflect autonomic nervous system functioning implicated in arousal, emotion (dys)regulation, and psychopathology. Psychophysiological variables can be leveraged to assess cognitive, social, and functional domains by overcoming subjective biases associated with self-, caregiver- and observer report. Psychophysiology can be measured across multiple contexts using various biosensing devices. Naturalistic biosensing can expand diverse participation in neurobiological research, by removing barriers of time and geographic distance required for lab-based assessments. Biosensing in clinical contexts also has the potential to provide peripheral physiological indicators of response to psychotherapeutic interventions. Measuring psychophysiology naturalistically and during psychotherapeutic sessions can elucidate mechanistic and causal processes. This Perspectives piece provides guideposts for scientists and practitioners to consider when selected biosensing devices/systems for clinical and research applications. Empirical evidence supporting the use of biosensors for valid and reliable psychophysiological signals—like electrodermal activity (EDA), photoplethysmography (PPG), and electrocardiography (ECG)—are reviewed in terms of the end-to-end user experience, accessibility of raw data, data quality, and data security. Finally, considerations of racial bias and variation related to biological sex in psychophysiological measures are discussed. The ultimate goal of this Perspectives piece is to inform expanded use of biosensing for the measurement of peripheral physiology to (1) improve understanding of naturalistic disease processes, (2) predict treatment response, (3) guide treatment progression, and (4) identify underlying treatment mechanisms for further refinement in clinical and research settings.

## Lay summary

This work explores the growing use of biosensing devices/systems to track changes in bodily states that may map onto mental health phenomena. It provides guidance for practical use of biosensors in research labs, treatment settings, and every day contexts. By providing fundamental guidance, the field can better focus on improving reliability and accuracy of biosensors while also considering user experience and diversity in design.

## Introduction

“To be measured with high validity, psychopathology must be observed within the dynamic contexts in which symptoms manifest” [1]. This framework implores the field of psychiatry to expand outwards from traditional self-report assessments in non-naturalistic environments and tasks that are not evocative of real-world experiences and contexts. Biosensing in laboratory, clinical, and naturalistic settings can aid us in getting closer to validity of constructs of interest related to psychopathology with expanded inclusivity overcoming geographic, transportation, and timing-related barriers. As an objective measure, biosensing may also overcome reporter bias inherent to self-report and interview methods. Compared to other objective measures proposed for studying psychopathology (e.g., neuroimaging; blood- or saliva-based biomarkers), biosensing stands as a more cost-effective and mobile option. Here, we provide guidance on selecting biosensors for researchers and clinicians aiming to leverage physiology as a unit of analysis in their work.

## What is biosensing?

Physiology is a unit of analysis that can assess domains (e.g., negative and positive valence; arousal/modulatory systems) relevant to mental health phenomena [2]. Biosensing uses devices or systems (biosensors) to measure physiological signals that reflect dynamic behaviors and emotions. Observed behaviors and emotional states are underlain by measurable physiological processes [3]. Physiological phenotypes measured through biosensing can serve as potential indicators of risk for psychopathology, resilience, and treatment response [4, 5]. Biosensing can also aid in treatment optimization, by tracking whether a patient is responding or not to treatment. Biosensing can be used to actively or passively measure physiology in laboratory, clinical, or naturalistic settings, during resting, task, therapeutic, or other related conditions. As a result, biosensing has been used to identify biological fingerprints of mental illness (e.g., anxiety disorders, depression, posttraumatic stress disorder) and mechanisms underlying interventions like exposure-based cognitive behavioral therapy.

## Why use biosensing?

Many researchers and clinicians in the mental health space may be interested in two main constructs: arousal/reactivity and regulation. Vulnerability to negative health outcomes may be driven by individual variation in neural pathways critical for emotion regulation, attention, memory processing, and other cognitive functions [6, 7] as a result of genetic/epigenetic and environmental factors (e.g., exposure to trauma and adversity). These neural pathways modulate arousal/reactivity and regulation, and they are modifiable with treatment [8]. Given that individual differences in arousal/reactivity and regulation map onto research domains of interest to psychiatry and mental health (e.g., arousal/regulatory, positive valence, negative valence) [2, 9, 10], using biosensing to measure arousal/reactivity and regulation could serve as a means to objectively predict risk, resilience, and treatment responsivity. Electrodermal activity (EDA; signal from which skin conductance level and skin conductance response can be derived), heart rate (HR; derived from electrocardiogram—ECG—or photoplethysmography—PPG), and temperature can serve as indicators of arousal/reactivity. Heart rate variability (HRV; derived from ECG or PPG) is a common indicator of regulation.

Biosensing has multiple intersectional clinical and research applications. EDA in the immediate aftermath of trauma, for example, has been identified as a replicable predictor of severity of posttraumatic stress symptoms and odds of diagnosis 6 months later [11, 12]. Another example of biosensing comes from the exposure therapy literature. Effectiveness of exposure-based therapies relies on sufficient activation of the representation structure of the feared stimulus or scenario. For example, imagery, narrative, or sensory stimuli can evoke the same physiological output as is observed during the actual stressor or as was observed at the time of the original precipitating event [3, 13]. Therefore, physiological responses can serve as an indicator as to whether the stimulus or scenario used to evoke the representational structure of the stressor or precipitating event is sufficient in doing so [3, 13]. This is essential to making meaningful change through the therapeutic process of exposure. Physiological state can then be monitored during the exposure to track whether habituation occurs, that could indicate appropriateness for termination of the exposure, or to identify relations between self-reported subjective units of distress and physiological state. Finally, over the course of treatment, physiological measures could be used as indicators of changes in arousal and regulation (e.g., decreased arousal, increased regulation) that may correspond to treatment response or nonresponse. Biosensing could also be used to inform treatment selection based on objective physiological state in stratified clinical trials or in precision medicine frameworks.

## Selecting biosensors

Henry and colleagues [14] recommend a 5-step selection process originally developed for choosing an ecological momentary assessment platform that can be adapted for biosensors: (1) create an individualized and prioritized list of features that you will require; (2) research candidate devices/systems and select a few that meet your needs; (3) connect with the Institutional Review Board or data security officer at your institution/practice to determine technology and privacy requirements; (4) meet with developers to walk through features and specifications; and (5) request and conduct free testing/pilot data collection to ensure needed capabilities are in place and detect any user ‘bugs’. To guide this process, we provide a Biosensor Checklist (see Appendix 1) and provide a series of considerations for selection below.

### Construct(s) of interest

First, users must determine their construct(s) of interest. The construct of interest will dictate the biosensor(s) required. For example, a researcher interested in observing changes in arousal during exposures may want to measure heart rate, thus requiring electrocardiography (ECG) or photoplethysmography (PPG) sensors. Identifying multiple indicators can be of benefit to verify proper collection/recording and to serve as a backup in case other measurements fail. In this example, temperature within physiologically-plausible ranges may be used to ensure that ECG or PPG sensors had good connection with skin and data were being properly recorded. EDA could serve as a backup for or convergent validator of HR; HRV could also serve as a divergent validator for HR.

### Data collection context(s)

The context in which data will be collected must then be specified. Where will measurements be obtained—in a lab, in a clinic, or in naturalistic settings? Location of data collection will dictate the types of biosensing devices/systems that may be appropriate for the research design through considerations of ease of use, training needs, battery life, and wired versus wireless systems. For example, wearables like rings, watches, and user-friendly sensors or chest bands that can integrate with smartphones or tablets are feasible for use across settings—including naturalistic settings like homes, schools, and occupational settings where the participant/patient or their caregiver may be tasked with setting up the biosensor themselves. For naturalistic and clinical settings, biosensors that can be easily set up with minimal user training and are wireless are likely most appropriate. However, more complex platforms that require specialized recording software, integration with additional computing systems, and more precise placement of electrodes may not be suitable for naturalistic settings or for clinical use where time and training opportunities are limited. Location of data collection will likely also relate to whether data are being collected during a task or therapeutic session, or continuously in a passive manner. If data are to be collected during a task or therapeutic session in a clinic or lab setting, long-term battery life may be less of a concern, while sampling rate may be a greater concern. If researchers or clinicians are interested in event-related task designs or evaluating moment-to-moment changes during therapy, a higher-frequency sampling rate would be necessary for biosensors. In the same vein, users should also ensure that raw data will be made available, as summary data may average over seconds or minutes, losing the fine-grained temporal resolution that certain designs require. On the other hand, data collection in naturalistic settings may not require fine-grained temporal resolution and may be more permissive for devices with lower-frequency sampling rates. Naturalistic settings will require devices or systems with longer battery life. Some devices turn off at night to conserve battery life, so if sleep-related metrics are of interest, a device that can stay powered on continuously will be needed. Similarly, some devices will only sample data periodically—if data need to be available from specific timepoints or events, then a device that continuously samples data or allows users to prompt sampling would be required. Data storage should also be considered for naturalistic data collection—is WiFi needed to push storage updates to a cloud, or can the device store sufficient amounts of data until an upload can be pushed by the researcher or clinician if the participant/patient does not have access to WiFi? Finally, if built-in user support is not included in license agreements to aid patients, participants, or clinicians with setup and troubleshooting, researchers or clinical experts should develop support materials to share with users.

### Verification and validity

Verification, analytic validation, and clinical validation should all be taken into account when selecting a device or system [15]. First, verification—does the sensor capture data accurately and output data within a physiologically plausible and acceptable range? Second, analytic validation—do algorithms for noise filtering, artefact correction, and scoring of raw data function properly? Are the resulting metrics stable and accurate? These first two parameters are typically the top considerations when developing a wearable, but clinicians and researchers should still do their homework and identify data supporting verification and analytic validation for the biosensors they select. Importantly, clinicians and researchers should examine whether biosensors have undergone verification and analytic validation in diverse populations, to ensure that sensors are optimized for all skin tones and textures. Previous research has indicated lack of generalizability of psychophysiological data due to disproportionate removal of ethnoracially diverse individuals due to low or no response on certain biosensors (e.g., EDA sensors [16]). Researchers conducting clinical validation studies should be sure to include demographically diverse samples (e.g., ethnoracial identity, sex, age) and may choose to select multiple types of sensors to examine validity and reliability. While more expensive, many research-grade devices have FDA approval and may have more evidence supporting clinical validation and reliability. Of note, FDA approval is not always given at the device level—some devices/systems hold FDA approvals for one or two biosensors, but not all within the same device or system. If multiple devices are needed to distribute to multiple patients/participants simultaneously, a more user-friendly and cost-effective consumer-facing device might be more appropriate. With these parameters in mind, a device or system can then be selected.

## Applications

### Optimizing data collection

To ensure validity and reliability is maintained within and between biosensing experiments, appropriate and thorough training of users is required—whether it is a clinician, researcher, patient, or participant. Individuals conducting the setup of biosensors should be provided with a manual, especially if patients/participants will be doing the setup on their own outside of lab or clinic. Video demonstration can supplement written manuals, as 3D imagery may assist with the 3D nature of biosensing devices/systems. A see one, do one, teach one approach is recommended. Importantly, users should be educated on where biosensors are on the device and how they function. For example, educating a user about the distribution of eccrine glands on the palmar surface can help a user better understand the appropriate placement of EDA sensors.

To optimize data quality, we recommend the following procedures when using biosensors:

1. Clean skin appropriately prior to application. This may include washing with soap and water; for some sensors—like for EDA—clean the skin with non-alcohol based cleansers, as alcohol may dry out the skin and suppress the EDA measurement. Most devices/systems include cleansing instructions in their manuals, for both the user and for sanitizing biosensors after use.
2. Remove hair at point of contact between skin and sensors, if possible, to reduce interference with sensors.
3. Ensure good contact with the skin—for some sensors, this could be enhanced by using gels specific to the sensor. Good contact without excess pressure is important. Be mindful of placement such that sensors are not making contact with bone.
4. Reduce movement of sensor(s) against the skin. Taping down the ends of electrode leads or placing a sweatband over a wrist-worn device can help reduce movement and ambient light interference.
5. Reduce other interfering signals—users should remove any other biosensors that are not being used in the experiment. Users should also remove any jewelry or other objects that may touch or interfere with the sensors.

Researchers and clinicians may also consider monitoring environmental conditions like room temperature and humidity, as well as holding variables that may influence physiology constant—e.g., asking participants/patients to fast from caffeine for at least 2 h prior to assessment, collecting data at similar times of day where possible to account for circadian variation, etc. While it may not be possible to modulate medication use, researchers and clinicians should be aware of and possibly covary for medical conditions and medication use, particularly stimulant and anxiolytic medications.

### Analytic considerations

Researchers and clinicians should talk with developers and carefully read device manuals to understand any preprocessing that may take place prior to data output. Some devices/systems have proprietary algorithms that do initial artefact correction and filtering. Other devices/systems only provide summary data generated using proprietary algorithms, such that access to raw data is restricted. If preprocessing one’s own raw data, there are a number of free, open source software available through GitHub (e.g., LOTUS [17]; the Digital Biomarker Discovery Project [18]), R packages (e.g., psyphy [19]), and python toolboxes, as well as paid/licensed software (e.g., Kubios [20]; LedaLab for MatLab [21]; Mindware) that typically include more user-friendly GUIs. Pipelines should include steps for handling missing data and motion artefacts (if not already handled by proprietary device algorithms) and low-pass and high-pass filtering. Psychophysiological data from different biosensors can be aligned, as can task or clinical events, based on timing parameters (most devices output data with Unix timestamps that can be aligned with task timestamps or clinical events from audiovisual recordings or manual event stamping). Psychophysiological data may need to be log-transformed or require use of non-parametric tests given the often non-normal distribution of the data. Multilevel models, linear mixed effects models, or latent models are commonly selected for analyses given the nested structures of data and intensive repeated measurements over time. Given the multiple forking paths in the aforementioned analytic pipeline for psychophysiological data, it is always recommended that users preregister their analytic plans and, if not conducting exploratory research, their hypotheses.

### Consideration of sex and ethnoracial identity

The hypothalamic pituitary adrenal (HPA) and sympathetic medullary adrenal (SMA) axes that underlie psychophysiological responses are closely interconnected with the hypothalamic pituitary gonadal (HPG) axis. Thus, individual variation in psychophysiology may be, in part, attributed to circulating concentrations of sex hormones like estrogen, progesterone, and testosterone that map onto observed sex differences. While sex as a biological variable should be considered as a covariate in analyses leveraging psychophysiological data, corresponding measurements of circulating gonadal hormones would provide more nuanced understanding of factors associated with individual variation in psychophysiological responses. Where possible, this approach to measure gonadal hormones directly could not only better tap into mechanistic features of observed sex differences, but also better represent sexually-diverse individuals (e.g., transgender individuals, intersex people) in this realm of science. For developmental research in particular, this approach, or at least the measurement of pubertal status, may be all-the-more important to capture differences/changes in psychophysiology across sensitive periods [22].

Race and ethnicity, or ethnoracial identity, have commonly been used as proxy variables for discrimination, socioeconomic status, cultural differences, and genetic ancestry. Race is a social construct, not a biological variable, however experiences of racism may have dramatic impacts on one’s biology—including psychophysiological responses. For example, while some literature has hypothesized skin tone or texture as a factor contributing to individual variation in psychophysiological responses like EDA, more recent studies have identified structural inequities as the cause of this observed variation [23]. Therefore, researchers and clinicians should not simply examine ethnoracial status but also consider measuring discrimination, structural inequities, and other factors that could account for observed differences in psychophysiology.

## Conclusion

Biosensing is a great tool for real-time assessment of [psycho]physiology in lab, clinical, or naturalistic settings and offers insightful opportunities to advance understanding of mechanisms underlying psychopathology, behavior, and response to treatment. Numerous biosensing devices and systems are currently on the market—both consumer-facing and research grade—and choosing between options can pose challenges to researchers and clinicians. It is important to highlight that there is no perfect tool, and rather the individual needs of the user—particularly constructs of interest and contexts of use—can dictate selection. With the guidance provided herein, as well as the strategies for data collection and analytic optimization with considerations for sex and ethnoracial identity, users can more confidently integrate biosensing into their research and clinical programs.

### Citation diversity statement

The authors have attested that they made efforts to be mindful of diversity in selecting the citations used in this article.

## Supplementary information

Biosensor Checklist
