# Supplementary material for: Leveraging biosensors in clinical and research settings: a guide to device selection
Source: NPP Digit Psychiatry Neurosci. 2025 Jul 2;3:17. doi: 10.1038/s44277-025-00037-w (PMC12624877; doi:10.1038/s44277-025-00037-w)
Supplement: Supplementary file 1 — Biosensor Checklist [file 44277_2025_37_MOESM1_ESM.pdf]

Checklist for Selecting a Biosensing Device or System

Developed by Dr. Lana Ruvolo Grasser

Construct of Interest

- [ ] Arousal: Electrodermal Activity
- [ ] Arousal: Heart Rate
- [ ] Arousal: Temperature
- [ ] Arousal: Startle
- [ ] Regulation: Heart Rate Variability
- [ ] Movement: Actigraphy
- [ ] Other

Collection Context

- [ ] Laboratory
- [ ] Clinic
- [ ] Naturalistic
- [ ] Event-Related Task Design
- [ ] Block Task Design
- [ ] Continuous Collection (Awake only)
- [ ] Continuous Collection (Sleep and wake)
- [ ] WiFi Availability for Data Uploads

Priorities for Researcher/Clinician

- [ ] Cost
- [ ] HIPAA Compliance
- [ ] Compliance with IRB/Data Security Office
- [ ] FDA Approval
- [ ] Access to Raw Data
- [ ] SDK Integration

Priorities for User:

- [ ] Ease of setup / use
- [ ] Comfort
- [ ] Privacy settings
- [ ] User support availability
- [ ] Language and accessibility features
